# It’s about being the good parent: exploring attitudes and beliefs towards active school transportation

**DOI:** 10.1080/22423982.2020.1798113

**Published:** 2020-07-22

**Authors:** Hanna Forsberg, Stina Rutberg, Katarina Mikaelsson, Anna-Karin Lindqvist

**Affiliations:** Department of Health Sciences, Luleå University of Technology, Luleå, Sweden

**Keywords:** Active school transportation, attitudes, children, health behaviour, parents, physical Activity

## Abstract

In recent decades, there has been a decline in active school transportation (AST). Parents play an important role as the key decision-makers of children’s AST, and there is a need of more knowledge about the decision-making process and parents’ beliefs towards AST. The overall aim of this study was to explore parents’ attitudes and beliefs towards AST in the northern part of Sweden. Twenty parents participated in semi-structured interviews, which was based on the theory of planned behaviour. Qualitative content analysis was used to analyse the interviews. The analysis yielded one main theme, “Parenting and active school transportation – making route choices in a changed landscape” and four subthemes, “Knowing that it is beneficial while struggling with daily life”, “Considering barriers and solutions to enable AST”, “Parenting is challenging and about balancing”, and “Reflecting and contemplating about what we and others do”. Winter conditions affect parents’ decisions, and this needs to be considered when facilitating AST in these regions. Overall better health, increased physical activity, time spent outdoors, and free play were revealed as positive outcomes of AST. Decisions were also influenced by social norms and how the idea of parenting has changed through generations. The findings of this study are likely to be important when promoting AST.

## Introduction

Studies have estimated that about 80% of all children do not achieve the recommended 1 h of moderate to vigorous physical activity per day [1,2]. Among the Nordic countries, Sweden has the lowest levels of daily physical activity among children [1]. Unfortunately, during the last decades, the number of children who walk or bike to school has decreased as well, also known as active school transportation (AST) [3]. Furthermore, children who use AST are more physically active, and AST has, therefore, been suggested as an opportunity to increase physical activity [4,5]. From a behavioural perspective, evidence indicates that lifelong patterns of physical activity are established in early childhood, highlighting the importance of promoting physical activity in the early phases of life [6]. During childhood, parents are among the primary influencers regarding children’s physical activity and serve as the key decision-makers of AST [7,8]. To understand what affects parents’ decisions, several studies have been conducted, identifying their perceived barriers to AST [9]. The built environment, traffic-safety, distance, crime-related safety, and social support have frequently been cited [9]. However, parents who drive their children perceive the environment as more dangerous, and other findings from intervention studies indicate that regular use of AST might decrease parents’ perceived barriers [10,11]. Furthermore, Westman et al. found associations between parent’s reasons for driving their children to school and social convenience, showing that mode choice was mostly determined by practical reasoning and that parents wished to spend time with their children during travel [12]. Contrary to previous research, these authors found no associations of mode choice with safety or distance [12]. Taken together, this research displays an overall complex picture of parents’ decisions, involving both personal and environmental aspects [13]. However, studies addressing AST are suggested to ground their investigations in theoretical foundations [14], ensuring to target the right determinants of behaviour. To understand, explain, and predict active transportation and health behaviour, the theory of planned behaviour (TPB) has previously been used [14–16]. The model suggests that behaviour can be predicted by intentions [17]. Intentions, which represent the willingness to perform a certain behaviour, are, in turn, related to three psychological constructs; attitudes, subjective norm and perceived behaviour control [17]. Attitude refers to an overall positive/negative evaluation of the behaviour. Subjective norm concerns beliefs about the perceived social pressure to perform/or not to perform the behaviour, and perceived behaviour control reflects beliefs about the ability or difficulty of performing the behaviour. Overall, the TPB model has been performing well across various health-related behavioural categories to explain intentions, including physical activity [16,18]. Furthermore, the majority of studies regarding parents’ influence on children’s AST has been conducted in the USA, using quantitative methods [9,14]. The importance of exploring psychosocial factors in different countries, such as in Europe, has been stressed, since AST varies widely across different cultures, economies, and geographical locations [9,14,19]. Finally, there is still little knowledge about how cold climates, such as in the Nordic countries, affect AST. Therefore, in this study, we aimed to address the limitations mentioned above, as we explore parents’ attitudes and beliefs towards AST in the northern part of Sweden.

## Methods

### Design

Since the aim of the study was to explore parents’ attitudes and beliefs towards AST, a qualitative design was chosen [20].

### Context

The study was part of a larger ongoing research project, aiming to promote AST [21]. The present study was conducted in a municipality in a northern county in Sweden. Approximately 80,000 inhabitants reside in the municipality, which is characterised by both rural and urban areas. The northern part of Sweden has long cold winters, reaching sub-zero temperatures that can last for months, and short springs and summers [22]. Because of the region’s proximity to the Arctic Circle, daylight is limited to only a few hours during the winter season, while the opposite occurs in the summer, with a few hours of darkness [23].

### Procedure and participants

In the present study, six principals were asked to share information about the study with parents’ at their schools, of which five agreed to participate (one principal of an independent school and four principals of municipal schools). In the information shared with parents, both users and non-users of AST were encouraged to participate in the study. If parents were interested in participating in the study, they were asked to send an email to the first author. The parents decided the location and date for the interview. Eleven parents were recruited from the municipal schools and nine from the independent school. The included schools socioeconomic index varied, and the municipal schools were located in both rural and urban settings. The independent school was located in an urban setting. In total, sixteen mothers and four fathers, aged 35–53 years who had children in primary school (grade 1–6), agreed to participate. Parents’ educational level varied; seventeen had a university degree, and three had graduated from upper secondary school. Parents estimated the children’s distance from home to school or a bus stop, which ranged from 100 m to 4 km, with an average distance of approximately 1.65 km. Two parents reported their children not using AST at all; two parents reported that some of their children used AST. Seven parents reported their children using AST based on the season, and nine parents reported their children using AST the whole year, with a few exceptions due to severe weather and season conditions.

### Data collection

The data was collected through individual semi-structured interviews, using an interview guide [20]. The interview guide was inspired by the framework of the TPB [17], and covered three areas; 1. Parent’s positive and negative experiences and reflections about AST: 2. Parents’ experiences and beliefs about other persons influencing decisions regarding their children’s use of AST, and finally: 3. Parents’ beliefs about barriers and facilitators regarding AST. Follow up questions were used to get more in-depth answers. One pilot interview was conducted by the first author to test the interview guide, and discussions with co-authors resulted in minor revisions. The interviews were conducted during spring 2019 and took place in a quiet room either at their home (2), workplace (9), or an office at the university (9). The interviews lasted between 18 and 74 min and were recorded and transcribed verbatim.

### Data analysis

Qualitative latent content analysis with an inductive approach was conducted, inspired by Granheim and Lundman [24]. The process of analysing data started with the first author listening to the recorded interviews, and reading when transcribed verbatim. In the next step, guided by the aim of the study, the whole text was divided into meaning units. The meaning units were then condensed and coded into preliminary categories. During this process, all co-authors discussed the codes and preliminary subthemes. In this phase, the authors strived to stay close to the text. By going back and forth between meaning units, codes, and categories, the categories were summed into one main theme and four subthemes.

### Ethics

Before the interviews, participants were given oral and written information about the study, including confidentiality and their right to withdraw participation at any time without explanation [25]. The regional ethical committee in Umeå approved the study (Dr 2018–10-31 M).

## Results

The analysis yielded one main theme and four subthemes (Table 1). Representative quotations from mothers (M) and fathers (F) were used and were labelled with a participant number (1–20).

**Table 1.** Overview of the results.

Theme	Parenting and active school transportation – making route choices in a changed landscape			
Subtheme	Knowing that it is beneficial while struggling with everyday life	Considering barriers and solutions to enable AST	Parenting is challenging and about balancing	Reflecting and contemplating about how we and others do

### Parenting and active school transportation – making route choices in a changed landscape

The interviews revealed an overall complex picture of parents’ attitudes, beliefs, and decisions concerning AST. The parents considered aspects that ranged from benefits of AST to fear of their child being hurt and, at the same time, contemplating the role of parenting, compared to other parents’ acting and their own upbringing. A sort of balancing act between overprotectiveness and not exposing children to unnecessary risks was described, illustrating parents’ choices in a changed landscape. The subthemes describe these aspects in more detail.

### Knowing that it is beneficial while struggling with everyday life

Parents acknowledged physical activity in general as beneficial to children and important since they believe they spend too much time engaging in sedentary behaviours. As a result of AST, parents recognised benefits such as the increased amount of physical activity in everyday life for their children, contributing to overall better health (specifically strength, coordination, and fitness, as well as reducing the risk of overweight). Physical activity was considered essential, and parents described childhood as a time for building their bodies and laying the foundation for the rest of their life.

*“I don’t really think children need to be in sports, the important thing is that they are outside playing and activating, and they can do that a lot by themselves, or when they commute to and from school”* M11

The positive effects on the environment were also considered as a benefit from AST (e.g., reduced exhaust gases, which were thought to be beneficial for sustainability). Parents reflected on how this, of course, was dependent on them not using the car. The combination of health and sustainability were described as the most important outcomes of AST. Being outdoors was also described as a benefit related to AST, which was thought to reduce time indoors and engagement in sedentary activities. By being outside on their way to or from school, parents noticed that the total time children spent playing outside was extended. Therefore, parents considered AST as an opportunity for children to be outdoors, getting fresh air, daylight, and being in contact with nature. The parents also thought that the benefits of physical activity and being outdoors might contribute to children’s ability to concentrate during the day and being more alert and ready to learn in school, thereby improving school achievement.

*“I think that there are huge benefits for all children to be physically active before school starts, they can get rid of some energy and wind down, they would have time to reflect before they get to school, if they walk with friends, then there would be time for them to vent and chat and if several more walked, they would be able to focus faster when arriving at the classroom”* M2

Walking or cycling together, either with friends or siblings, was also regarded by parents as an important factor for motivation and making it more fun. They also thought it was more common for children who used AST to follow each other home after school to play. Playing when travelling to and from school was considered a positive outcome of AST, and as an opportunity for children to use their imagination. Some parents even thought that their children would not play (to the same extent) with children not transporting actively in general. Parents also thought learning to be responsible and independent was important for children, and how many tasks related to AST were in line with their development. This was mainly related to how children handled being in traffic but also other specific tasks, such as being responsible for getting ready and being on time for school and choosing the appropriate clothes. AST also fostered skills, such as responsibility over the door key and being alone at home (either in the morning or in the afternoon). The independence of the children was also thought to be a significant benefit for the parents, since they did not have to spend time engaging in their children’s transport.

*” … for the sake of children’s independence, they need to learn not to be dropped off and picked up as in preschool, they are getting older now and need to actually take some own responsibility transporting to and from school”* M12

The parents also described how children appreciated having the possibility to go home when they wanted after school finished, not being dependent on parents driving them, which also resulted in shorter days. Nevertheless, time constraints in everyday life were regarded by some parents as a disadvantage to AST, referring to the time limit in the morning and afternoons during weekdays. Dropping children at school on their way to work was described as a way to save time. Some parents also explained that if they were driving one of the younger siblings to school, the older ones were often offered to ride along. Some thought mornings were most stressful, while others also described the afternoons as problematic since their children often had sports activities after school. Having more than one child could mean that schools and sports activities were placed at different locations, making it impossible to arrive on time without the car. Using the car was, therefore, considered as more convenient and as a way to optimise daily life, and seen as a way to be able to shop for ingredients for dinner, have time to prepare it, and transport children to and from school and to various activities on time.

*“It’s faster, and we get to sleep longer in the mornings and also in the afternoons, when there are sports practices and activities immediately afterward, then it’s about bringing all the stuff needed to be packed in the car, it can be clothes for horseback riding, football, and basketball, then picking up the children, bringing snacks … It’s about convenience, that’s the reason why I choose the car, it is faster, more flexible and results in less time spent on the transport itself”* M17

### Considering barriers and solutions to enable AST

The parents thought that the degree of safety along the way to school was very important to be able to let their children walk and cycle on their own in traffic. Barriers regarding traffic along the way to school varied. High-speed roads were generally perceived as dangerous, especially if there were no separate cycle lanes. This was enforced by a sense of feeling that motorists were not always showing respect to cyclists and pedestrians. Parents also experienced that motorists were using high speeds nearby schools, which was perceived as particularly problematic. Some also argued how this created a vicious circle, since protecting children from risks due to traffic, in fact, increased traffic.

*“many parents drive their children, and whenever you go there, it is very, very busy in the schools’ parking zone, the number of cars driving is hazardous for children who are actually walking … ”* M13

To enable AST, parents considered efforts to decrease traffic nearby and in the school area to make it safer for children. Other risks mentioned were unsupervised crossing points and intersections with heavy traffic and incorrect road signage. The parents also reasoned that a longer distance could be related to increased risk, since it enhanced the number of unsupervised crossing points for children to pass. To enable AST, the parents, therefore, thought it was important to build and construct cycle lanes, making an effort to make them safe and separated from other traffic, especially at crossing points. Beyond traffic, parents also worried about the possibility of children encountering unpleasant strangers along the way to school. Parents felt safer if their children joined siblings or friends along the way or by getting some kind of confirmation of safe arrival through telephone contact. Some also reasoned that by driving them to school, they could watch their children walk inside the school, and not need to worry.

*“If I wouldn´t be able to know if they had arrived safely to school, then I wouldn´t be able to relax at work, I need some kind of confirmation to be able to unwind, you know, ok now they are at school, now I can breathe out”* M9

Regarding winter, parents also expressed concerns about the large amounts of snow gathering at crossing points from snowplowing, making the children difficult to see. They also described how in periods of winter, there might be a lot of snow on the cycle lanes, making it slippery and heavy. Maintenance of cycle lanes was, therefore, mentioned as important to enable AST, such as ploughing and sanding during wintertime, and taking away gravel during springtime. Parents also felt that good street lightning was important due to the long period of darkness during winter. Related to this, how children used AST over the year varied somewhat by parents’ descriptions; some parents stated that, during wintertime, they drove their children to school by car, referring to cold weather and overall bad conditions for cycling.

*“During winter there are enormous amounts of snow gathered in great piles, at many crossing points, so basically you do not see if there is anyone at the crossing before you arrive with the car close up, so there are great risks in the winter”* M10

However, according to some parents weather was not necessarily a barrier to AST, as long as it was not heavy rain, ice-cold outside, or snowstorms. Thus, parents thought it was important to provide children with the right equipment to enable AST, such as a suitable bike and appropriate clothing. Some parents reflected upon how this could sometimes become an economic issue, but at the same time reasoned that it did not have to be about buying the most expensive equipment and suggested choosing cheaper options available, such as a second-hand bike. However, to enable cycling for all children, some parents thought that the municipality could take some responsibility and arrange service days, helping children to maintain their bikes. To further facilitate AST, parents also suggested that the municipality should encourage citizens through information and campaigns. Besides the municipality, parents also emphasised the importance of schools promoting AST by talking to and teaching children about it. Regarding children, parents thought it was important that schools made it fun and motivating.

*“I think they should talk a lot about it in school, about the importance of being physically active, so it becomes legitimate to cycle”* M4

Age was another important factor mentioned by parents since it was related to skills like being able to calculate for consequences of actions, peripheral vision, and sense of orientation. Age was also considered by parents as an important factor related to stamina, reasoning that younger children could easily get tired and exhausted, while older children had better prerequisites for managing distance. Parents referred to different ages being suitable for handling traffic and managing AST. Some parents thought that children would be capable of AST at 12 years old, when most of the required skills were developed. Others reasoned that the age of 8 and 9 years could be appropriate, while 6-year-old children were considered too young by some. However, because of individual differences in children’s development and readiness, it was considered important to estimate and asses children’s skills and abilities, which was generally conducted by following and observing them in traffic. This could mean going to a specific place, identified as dangerous, the parents then instructed children about were to be observant of risks and where they needed to stop and get off the bike. This also included finding the best way to school with the least risks and traffic. The parents also reasoned that they sometimes might be underestimating their children’s ability, and some also thought it was necessary to loosen their restrictions while learning to trust the child.

*“ … we are familiar with the road to school, and there is a place where you really need to be observant which we have informed her about, but then again you can’t be worried about it all, because then you wouldn’t dare to fly or drive on the highway either, we are not like that, however, before we give them permission to cycle, we look for the risks and talk about it, increasing her awareness, then she’s good to go”* M3

### Parenting is challenging and about balancing

Parents considered their role as somewhat difficult, balancing between not being too overprotective meanwhile not exposing their children to unnecessary risks. The parents described how they wanted to be identified and appear as good parents, which was not always recognised as compatible with activities such as AST. The parents felt sometimes overwhelmed by current collective beliefs and values on how they should be a good parent, such as engaged, supportive, and in control of their children at all times. Some parents also struggled a lot with thoughts concerning what others might think, judging them for not caring enough, and being irresponsible when letting their children go walking or cycling on their own.

*“I have been reflecting a little bit about what it means to be a good parent. It’s a little bit about the values that lie beneath, if you let the children off on their own, you may be considered as somewhat irresponsible, if you want to be a responsible parent you’re supposed to be in control of your child”* M15

The parents also described how a lot of time was spent on children’s activities nowadays. This was perceived as positive, but only to a certain point, meaning that it sometimes merged into acting like “lawn movers” for their children. Being the parent in control and responsible could easily transform into actions such as driving children to school and other activities, and being present during sports practices. Some parents expressed that acting like “lawn movers” was also a way to care for their children and avoid risks. Others reasoned that walking or cycling with children could mean being a good parent instead of driving them. Parents also reflected on how they were acting differently in comparison to their own parents when they were children. They described how their parents usually did not drive them anywhere and that active transport was a common choice back then.

*”I think people, in general, drive their children a lot more than in former days, I get the feeling that parents act a lot more like lawn movers nowadays”* M1

Parents also thought that the distances that they used to walk or cycle when they were children are today considered too far and a reason for driving children. Likewise, differences were also identified in how parents engaged in children’s activities; in former times, they could not have expected their parents to be engaged and present all the time. Some parents thought that these generational parenting changes were related to collective beliefs and values, but also to an increased awareness of risks. Constantly hearing news about accidents involving children and terrible things happening made parents feel scared and stressed about the safety of their children. Nevertheless, some parents also saw it as a part of the technical developments of recent decades and an increased focus on preventing accidents and risk elimination.

*“When I think about the freedom I had as a child in contrast to the freedom I give my children, if they go to visit a friend, then they are obliged to bring their phones and call me to inform them that they have arrived. That wasn’t possible in former days, then you could only hope that everything had gone well, but I think we are more careful nowadays and more aware of the risks, but maybe we are just exaggerating it, but if you have the possibility to avoid something happening to your child, then you’re just glad to do it”* M10

Viewing changes from a positive perspective, parents recalled growing up in a world without seatbelts and bicycle helmets, which was identified as a significant improvement. As a result of development, specifically smartphones, parents described feeling more obligated nowadays to have control over things that were not possible before, as in controlling their children’s safety throughout the day with text messages and calls. Parents also considered the possibility of increased attention towards risks leading to an excessive negative illustration of safety and security in society. Some parents also reflected upon whether the perceived risks were legitimate or not. Parents also related AST as a part of the upbringing, feeling responsible for allowing them to inherit good habits. The parents thought it was important not only to give their children the right upbringing, but also to be role models and set an example, such as walking and cycling themselves.

### Reflecting and contemplating about how we and others do

Parents reflected and contemplated how other parents and children were using AST and how this affected themselves. They described feeling affected by observing what other parents were letting their children do. Watching other children manage to walk and cycle on their own motivated and encouraged them to let their own children do the same. Some parents also thought that other parents could act as role models for each other, sharing experiences and solutions related to AST.

*“there is this other boy who attends to the same school … and he started to walk and cycle to school by himself at a very early age, which we noticed and thought X is not ready for that at all, and she wasn’t because this boy was much more independent, which could be observed by the way he navigated in traffic, but, as a matter of fact, he became our motivation … and was probably contributing to why we let X go by herself now”* M9

Parents also thought that children could affect each other, reasoning that if one or a couple of children in the same area started using AST, several more would follow. Nevertheless, some parents recognised that some were driving their children often and over short distances. The parents described how this sometimes caused discussions with their own children, wondering why they could not get a ride as their peers. Some parents described how they approached this by motivating their children and explaining why it was important not to use the car all the time and how AST was positive for them. Therefore, some parents argued that if everybody would be using AST, then nobody would question it or discuss it, because it would be considered as the norm instead of driving. Related to this, parents, therefore, referred to attitudes in the whole society contributing to and normalising always using the car and making it a habit. Some also reasoned that this was not only about AST and needed to be understood from a broader perspective, including attitudes towards transport in general. The parents also reasoned that cycling had historically been perceived as a symbol of low social position. Meanwhile, a nice car represents wealth and high social position. Parents experienced this in their own workplaces, where discussions were generally not about which bike they had, but rather which car they were driving, generating peer-pressure.

*“Certainly peer pressure exists in some form, previously cycling has been associated with low status, and the car still represents status and something you can show off with, at least from my perspective, I mean at work, nobody talks about which bicycle they have, they rather talk about their cars, and it’s not the same status showing up with a bike as it is in a new car or a company car … ”* F6

When striving towards a higher social position in the lowest social classes, a nice car could symbol belonging to a higher social class, rather than the lowest class. Parents thought that individuals in the lowest social class did not strive towards using the bike or taking the bus, but instead strived towards a better and easier life, which the car might represent for them. Meanwhile, parents thought the development in the highest social classes had been the opposite. In contrast, privileged people with an organised life can afford to cycle to work for health and environmental reasons.

*“ … In the highest socioeconomic group, which also includes me in some way, I believe it is easy to say that I cycle to work for health reasons, but then you have reached a form of a privileged life, like unregulated working hours, high salary and a nice bike … ”* M14

## Discussion

In this study, we explored parents’ attitudes and beliefs towards AST, which to our best knowledge, is one of few such studies done in the Nordic context. We detected a somewhat complex picture, identifying several different aspects influencing the decision-making process and behaviour. Being inspired by the TPB framework, our results provide insights not only to barriers as reported by many studies in this area [9], but also the benefits and positive outcomes of AST, perceived by parents, such as overall better health, increased physical activity, ability to focus in school, extended time spent outdoors, and opportunities for free play. Identifying positive attitudes could be seen as equally important, since this is assumed to increase the likelihood of performing a behaviour [17]. However, Ajzen argues that although positive attitudes are an indicator of a greater likelihood of performing a behaviour, available resources and opportunities, such as time, will, to some extent, determine behavioural achievement [17]. In our study, some parents thought everyday life tasks were more convenient to accomplish by using the car, mostly due to time constraints, displaying how certain resources were affecting their decisions. Furthermore, our results not only confirm convenience as in previous studies [12,13], but also highlights the importance of understanding the prerequisite for modern family life when aiming to promote AST. For instance, ensuring that their children arrived in time for sports practice in the afternoons was one reason for not using AST. Previously, it has been concluded that children’s physical activity is associated with parental support, such as being assisted with transport to sports practices [26]. Also, this indicate that the choice of transport to school is not merely determined by the journey to school, but also by several other linked activities before and after school [27].

One barrier mentioned by some parents’ were winter conditions, which resulted in their children not using AST during this period. Building on previous knowledge about issues in the north regarding AST, these perceived barriers by parents are in line with studies showing a decline in AST during winter in Sweden, and similar seasonal differences have been found in Finland [28,29]. However, our results provide valuable information about parental beliefs regarding both barriers and facilitators related to AST during wintertime. This doesn’t only add knowledge in further understanding the decline, but also on how to promote AST during this period. This is likely to be important since wintertime has been targeted in efforts to increase AST in this region [28]. Some parents experienced uncertainty about the competence of their children, and one way to cope with the worry of using AST was to drive their children to school. These experiences might reflect changes that have occurred over a long period, as adults’ image of children in western countries has changed [30]. The transformation has moved towards perceiving children as vulnerable and dependent on their parents, and, as a consequence, they are given little independence [30]. This has been referred to as the bubble-wrap generation, illustrating parents’ wishes to protect their children from anything happening to them [31]. Also, to protect children, the decreased levels of children’s independent mobility has had unintended consequences, such as decreased everyday physical activity, lack of social skills, less use of imagination, and reduced self-efficacy and environmental competence [31,32].

Moreover, previous research has confirmed that the age of children is important regarding parents’ decisions about AST [15], which is also in line with our results. However, our results indicate a lack of consensus in parents’ reasoning about suitable ages for AST. Based on the parents’ explanations, it seems that the suitable age for AST can vary since it was perceived as something linked to the individuals’ competence. Parents described how they were aware of the individual differences regarding maturity and readiness among children, and they used strategies like estimating their skills in critical traffic situations. In Sweden, the national guidelines suggest an age-limit of 12 years, referring to the development of skills needed to navigate in traffic [33]. On the contrary, a recent report concludes a lack of evidence supporting the age recommendation of 12 years and, rather, stresses that it is a question of situation and context [34]. A fixed age recommendation could be misleading, since it might imply that an 8-year-old child with good prerequisites in the surrounding environment should not cycle, while a 12-year-old with bad conditions for cycling should. Also, the report highlights that the age recommendation only considers risks and not beneficial outcomes, which should be considered when designing recommendations [34], and is in line with the opinions of the parents in the present study.

Parents’ reflected how parenting had changed through the generations since they were children themselves. They expressed feeling pressured to be a good parent, which were associated with being responsible and in control all the time, and not letting their children go out their own. These results are consistent with previous research showing that parenting has changed during the last decades to involve more supervision and monitoring of children than in the past [35]. Furthermore, aiming to be a good parent resulted in a balancing act between not exposing children to unnecessary risks and being too overprotective. The increased attention towards risks was, by some parents, thought to be related to the vast flow of information now available, creating greater awareness of risks. Thus, a report shows that about 10% of all fatalities in Sweden are related to road traffic accidents; meanwhile 55% results from accidental falls [36]. Also, according to the same report, 65% of the public believes road traffic was the most common accident leading to death. The report concludes that the public’s perception of fatalities differs from reality and might reflect the media reporting, which is in line with parents’ reflections in our result. However, as concluded in previous research, parents’ concerns regarding safety should not be diminished and need to be considered when aiming at promoting AST [10].

The results also provide insights into how social norms affected parents’ decisions. For example, some parents described feeling unsure about letting their children use AST, but by observing other parents allowing it, made them feel more confident about doing so as well. Repeatedly, parents used other parents and their children as reference groups when contrasting their behaviour to others. In previous research, the subjective norm has been shown to have relatively limited influence on health behaviour, compared to attitudes and perceived behaviour control [16,37]. Kim et al. suggested that this might be due to researchers choosing irrelevant reference groups, and recommend that this could be further explored in interviews [37]. The results of our interviews suggest that other parents and their children are important reference groups to parents. Also, the authors conclude that the majority of studies have only measured subjective norms (social pressure to perform the behaviour), which might also have contributed to inconsistency, since when it comes to physical activity behaviour, descriptive norms (observing others) might be equally important [37]. Our results indicate that both subjective and descriptive norms might be of importance in understanding how social norms affect parents’ decisions, which is in line with previous studies showing that subjective norm was the strongest predictor regarding parents’ intentions to increase their children’s walking behaviour to school [38].

### Methodological strengths and limitations

We chose a qualitative approach, through individual interviews and latent content analysis, to explore parents’ attitudes and beliefs towards AST in Sweden. Considering trustworthiness is important, and according to Öhman [39], peer debriefing can increase credibility. The analysis was thoroughly discussed within the research team, and we moved between the theme, subthemes, and the interview text to ensure that we included all data in the study and that our interpretations were reasonable. We wanted to have a wide variety of participants to enhance the possibility of exploring parents’ different experiences and thereby strengthening the credibility of the study [24]. The included participants were mothers and fathers at different ages from both rural and urban settings, their children attended schools with varying socioeconomic index, and the parents had different prior experiences of AST. However, most of the participants had a university degree and were women, which might have influenced our results. Thus, in comparison with other interview studies conducted in this area, the participation distribution is quite similar [40]. Also, although Sweden is one of the most equitable societies in the world, women are still primarily responsible for the children, family, and household [41], which might be a reflection of the participant distribution in the present study. We used an interview guide comprised of a few main questions with follow-up questions, and both positive and negative experiences were revealed, which suggests openness from the participants. The results of the present study should be interpreted based on its context. One limitation of this study is, therefore, that it was performed in a small town, which might have influenced parents’ attitudes and beliefs. To facilitate transferability, the research process, the participants, and the results have been described in detail. Authors can offer suggestions concerning the transferability; however, as stated by Graneheim and Lundman [24], the transferability of the results to other contexts must be considered by the reader.

## Conclusions

Few studies have explored parents’ attitudes and beliefs towards AST in the Nordic context. The results of this study illustrate how seasonal changes, such as winter conditions, affect parents’ decisions, and this needs to be considered in order to facilitate AST in these regions. These results are also likely to provide valuable knowledge for practitioners and policymakers in other similar contexts outside Sweden. This study captured perceived barriers but also gained insights into the positive outcomes of AST experienced by parents’, such as overall better health, increased physical activity, time spent outdoors, and free play. This adds knowledge to aspects that have received little attention previously, considering the impact that positive attitudes have on behaviour. Another finding that affected parents’ decisions was the influence of social norms, the view of children’s competence as well as independence, and how the idea of parenting has changed through the generations. Subsequently, the knowledge gained from this study has practical implications in incentives aiming at promoting children’s AST.
